# Retraction: A Cross-Talk between TrkB and Ret Tyrosine Kinases Receptors Mediates Neuroblastoma Cells Differentiation

**DOI:** 10.1371/journal.pone.0267929

**Published:** 2022-04-27

**Authors:** The PLOS ONE Editors

After this article [1] was published, concerns were raised about similarities between some of the lanes in the western blot images in Figs 1, 2, 4 and 5 and, a region of similarity between two of the microscopy images in Fig 3.

Specifically, the following data appear similar:

- Lanes 2 and 3 in the GAP-43 LAN-5 panel in Fig 1B.
- Lanes 2 and 3 in the IB:pTyr SH-SY5Y panel in Fig 2B.
- Lanes 2 and 3 in the IB:pTyr SK-N-BE panel in Fig 2B.
- Lanes 2 and 3 in the IB:pTyr LAN-5 panel in Fig 2B.
- Lanes 1 to 3 and lanes 4 to 6 in the IB:TrkB panel in Fig 2C.
- The right half of panel I and the left half of panel J in Fig 3.
- Lanes 2 and 3 in the pRet LAN-5 panel in Fig 4A.
- Lanes 2 and 4 in the Ret SH-SY5Y panel in Fig 4A.
- The 72h SH-SY5Y alpha tubulin panel and the 18h SK-N-BE alpha tubulin panel in Fig 5A.
- Lanes 2 and 3 in the Ret SK-N-BE panel in Fig 5B.
- Lanes 2 and 3 in the Ret LAN-5 panel in Fig 5B.

In editorial follow up on these issues, underlying images from the original panels of concern were provided for Fig 5A. However, the *PLOS ONE* Editors consider that the underlying image provided for the 72h SH-SY5Y alpha tubulin panel of Fig 5A did not appear to match the blot shown in the published figure.

The corresponding author stated that the remainder of the original data for the western blots presented in this article are no longer available. The corresponding author provided image data from replicate experiments in support of results reported in Figs 1B, 2B, 2C, 3, 4A and 5B, but these did not resolve the concerns about the published figures.

In light of the above issues, which could not be resolved in the absence of the original underlying data, the *PLOS ONE* Editors retract this article.

LC and CLE stand by the article’s findings.

LC and CLE disagreed with retraction. VdF and AD could not be reached.

## References

[1] Esposito CL, D’Alessio A, de Franciscis V, Cerchia L (2008) A Cross-Talk between TrkB and Ret Tyrosine Kinases Receptors Mediates Neuroblastoma Cells Differentiation. PLoS ONE 3(2): e1643. 10.1371/journal.pone.0001643 18286198 PMC2242850

